# Brain white matter microstructure abnormalities in children with optimal outcome from autism: a four-year follow-up study

**DOI:** 10.1038/s41598-022-21085-8

**Published:** 2022-11-23

**Authors:** Manxue Zhang, Xiao Hu, Jian Jiao, Danfeng Yuan, Sixun Li, Tingting Luo, Meiwen Wang, Mingjing Situ, Xueli Sun, Yi Huang

**Affiliations:** 1 Mental Health Center, West China Hospital of Sichuan University, Chengdu, China; 2 The Second Affiliated Hospital of Chongqing Medical University, Chongqing, China; 3 West China Second Hospital of Sichuan University, Chengdu, China; 4 Brain Research Center, West China Hospital of Sichuan University, Chengdu, China

**Keywords:** Neuroscience, Psychology, Neurology

## Abstract

Autism spectrum disorder (ASD) is a lifelong neurodevelopmental disorder, with only a small proportion of people obtaining optimal outcomes. We do not know if children with ASD exhibit abnormalities in the white matter (WM) microstructure or if this pattern would predict ASD prognosis in a longitudinal study. 182 children with ASD were recruited for MRI and clinical assessment; 111 completed a four-year follow-up visit (30 with optimal outcomes, ASD−; 81 with persistent diagnosis, ASD+). Additionally, 72 typically developing controls (TDC) were recruited. The microstructural integrity of WM fiber tracts was revealed using tract-based spatial statistics (TBSS) and probabilistic tractography analyses. We examined the neuroimaging abnormality associated with ASD and its relationship to ASD with optimal outcome. The ASD+ and TDC groups were propensity score matched to the ASD− group in terms of age, gender, and IQ. TBSS indicated that children with ASD exhibited abnormalities in the superior longitudinal fasciculus (SLF), inferior longitudinal fasciculus (ILF), and extending to the anterior thalamic radiation (ATR) and cingulum; whereas the ASD+ group showed more severe abnormalities than the ASD- group. Probabilistic tractography analysis revealed that ASD+ group exhibited lower Fractional Anisotropy (FA) of the left superior thalamic radiation (STR L) than ASD− group, and that FA value of the STR L was a significant predictor of optimal outcome (EX(B), 6.25; 95% CI 2.50—15.63; p < 0.001). Children with ASD showed significant variations in SLF_L and STR_L, and STR_L was a predictor of ‘ASD with optimal outcome’. Our findings may aid in comprehension of the mechanisms of ‘ASD with optimal outcome’.

## Introduction

Autism spectrum disorder (ASD) is a neurodevelopmental disorder, characterized by deficits in social interaction, social communication, and restricted, repetitive patterns of behavior and interests^1^. The prevalence of ASD is estimated to be 1–2%^2^. With considerable long-term impairments across multiple domains^3^, the recommended treatment for ASD is behavioral training, while medication treatment utilized mostly to control comorbid disorders^4^. Currently, there is no specific treatment for the core symptoms of ASD.

The etiologies of ASD have been extensively studied^2,5^. ASD is regarded as a spectrum term that encompasses a plethora of possibly complex etiopathogenic processes^6^. Brain structure and function abnormalities have been implicated in the neurology of ASD^7,8^. The diffusion MRI (dMRI) study using tract-based spatial statistics (TBSS) analyses revealed that ASD is associated with several long fiber tracts^9,10^. The fractional anisotropy (FA), mean diffusivity (MD), radial diffusivity (RD), and axial diffusivity (AD) were all commonly used dMRI metrics. The most usually used metric was FA. Typically, a breakdown in WM integrity resulted with a decreased FA^11^. TBSS is a voxel-based methodology that provides a quick and automated means of assessing diffusion data^12^; nevertheless, determining a differentiation between adjacent, differently oriented fiber bundles with similar FA values is challenging, which results in an undesired loss of tract anatomical topology. Probabilistic tractography is beneficial for dealing with complex fiber architectures, such as crossing fibers and signal noise^13^. Previous tractography studies on ASD have shown abnormal WM integrity in the cingulum^14^, thalamus pathway^15^, and in the association fibers, including: superior longitudinal fasciculus(SLF)^16,17^, inferior longitudinal fasciculus(ILF)^18,19^, inferior frontal-occipital fasciculus (IFOF)^20^, uncinate fasciculus (UF)^20^. The breakdown in WM integrity typically generally resulted in a lower FA^21^, which is sensitive to fiber coherence, myelination, and fiber density. In addition, other dMRI metrics also measure axonal integrity and are sensitive to alteration of brain tissues and axonal damage^22^. Several meta-analyses reviewed the dMRI studies of ASD. One study found that ASD suffers from reduced FA in the right occipito-temporal, inferior occipital and lingual gyrus, fusiform and inferior temporal gyrus^23^; Another study indicated that the uncinate fasciculus(part of SLF) and frontal and temporal thalamic projections were responsible for complex socio-emotional functioning of ASD^24^; and the splenium of corpus callosum and the cerebral peduncle were found to be associated with ASD^25^. Above all, brain WM microstructure abnormality (impaired WM fiber integrity) has been confirmed in ASD; yet, no consensus on the exact tract has been obtained. Diverse ASD subgroups may exhibit distinct brain structural abnormalities, as well as distinct cognitive profiles, clinical profiles, and prognoses.

Individuals with a history of ASD who, over time, no longer meet the diagnostic criteria for ASD are referred to as ‘ASD with optimal outcome’^26,27^. The 'optimal outcome' was defined as the absence of significant autism symptoms and a normal range of social functioning; nevertheless, additional issues, such as impaired cognition or susceptibility to other mental diseases, may still persist. The developmental trajectory of children with ASD between the ages of two and fourteen years might be divided into six distinct categories, according to a large longitudinal study (n = 6975)^28^. The reported stability rates for ASD vary greatly, when socially adapted functioning is chosen as a criterion^29–32^. 3–25% of the children with ASD lose their ASD diagnosis later in life, according to Helt et al.^33^. The diagnosis of ASD remains persistent, and only a tiny proportion of children with a history of ASD have obtained the optimal outcomes, according to various longitudinal studies^26^. At the present, understanding about the mechanisms underlying the ‘optimal outcome’ is inadequate.

The neuroimaging disparity between ASD with current diagnosis and ASD with optimal outcome has revived renewed interest in retrospective cohort studies. According to one concept, 'neural normalization' can account for behavior normalization in ASD; in this scenario, the ASD with optimal outcome should resemble controls^34,35^. Alternatively, the concept of 'neural compensation' has been proposed, in which behavioral normalization is accomplished through the recruitment of alternative brain pathways rather than through the normalization of processing pathways. In this paradigm, brain activation in ASD with optimal outcome would be considerably different from control and current diagnosis ASD^36^. A reasonable assumption is the 'residual ASD' pattern, which shows that the ASD associated with optimal outcome continues to reflect the individual's ASD background. As a result, brain activation of ‘ASD with optimal outcome’ is comparable to that observed in ASD with current diagnosis^36,37^. Furthermore, we wondered whether ASD with optimal outcome exhibited distinct neuroimaging pathways from the outset in comparison to ASD with a persistent diagnosis? Previous longitudinal study found that processing speed abnormality of ASD in adulthood might partially attributable to WM microstructural integrity^38^. The anatomical asymmetry of the fusiform gyrus may have significant consequences for the severity of symptoms in ASD and demonstrates a developmental trajectory distinct from that of controls^39^. However, no cohort study has explored WM microstructure abnormality associated with ASD with optimal outcome.

The study aimed to explore the neuroimaging differences between ASD with optimal outcome, ASD with persistent diagnosis, and healthy controls. We wondered whether individuals who achieved optimal outcome exhibited a distinct WM microstructure, and whether this pattern might be used to predict clinical behavioral normalization. Our hypothesis were that (i) children with ASD had altered WM fiber integrity in the thalamus pathway, the association fibers, and other brain areas, as evidenced by previous studies; (ii) It was expected that the patients with optimal outcome would exhibit distinct brain WM abnormalities when compared to ASD with persistent diagnosis, and that the abnormalities would be associated with prognoses.

## Method

### Participants

The study was conducted from September 2014 to September 2021, with participants drawn from West China Hospital of Sichuan University and local community. The neuroimaging mechanism of ASD with optimal outcomes is analyzed as part of another study in this collection: The National Natural Science Foundation of China (NO. 81371495). The primary goal of the original research was to explore the biomarkers of ASD, and the current study is a follow-up study involving the same cohort. A total of 254 participants were recruited online and offline (ASD, N = 182; TDC, N = 72). Off-line method was conducted in the West China Hospital outpatient. Our online recruitment advertises were on the main social platforms including WeChat as well as the official accounts of the schools and hospitals involved. All patients were medication-naive and met the criteria of DSM-5. ASD subjects were excluded if they had a history of head injury, other neurodevelopmental or psychiatric disorders, and metabolic or genetic disorders such as Fragile-X Syndrome. TDC had never used psychotropic medications and had no history of neurodevelopmental, cognitive, learning, or neuropsychiatric problems. To confirm that they were typically developing, all of them had extensive testing, including the IQ and psychiatric testing. After a thorough explanation of the study, parents of participants provided the written informed consent for study participation, which was approved by the Medical Ethical Committee of West China Hospital of Sichuan University.

### Study process

**Figure 1:**
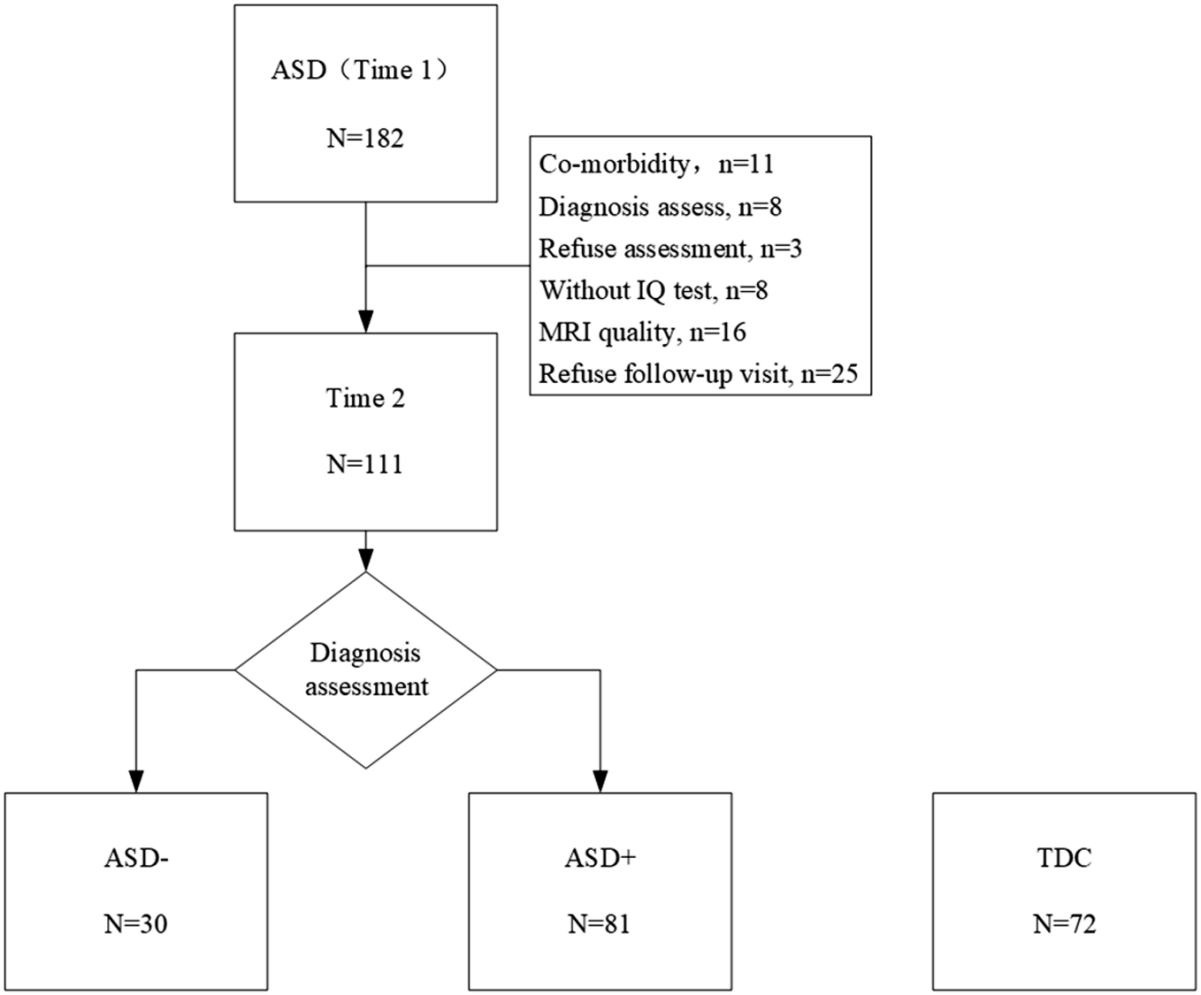
Study flowchart. *ASD−* ASD with optimal outcome, *ASD+* ASD with persistent symptoms, *TDC* typically developing controls.

All participants completed the diagnosis at time 1 (September 2014–September 2017): First, the child should be diagnosed by one professional child psychiatrist based on the DSM-5; second, a parent interview and a child assessment should be conducted using the Autism Diagnostic Interview-Revised (ADI-R^40^) and the Autism Diagnostic Observation Schedule (ADOS^41^) to confirm ASD diagnosis, as well as the Chinese version of the Schedule for Affective Disorders and Schizophrenia-Parent Long Version (K-SADS-PL) interview to exclude comorbidities; third, after receiving their diagnosis, participants were required to take an IQ test and have an MRI scan. Around four years after registration, participants were requested to take part in a follow-up assessment (time 2, September 2018 to September 2021). In Fig. 1, the workflow is shown. 111 participants completed the DSM-5 and ADOS assessments twice (ASD with optimal outcome, ASD−, N = 30; ASD with persistent diagnosis, ASD+, N = 81). 46 participants did not confirm to the inclusion/exclusion criteria at baseline; 25 children refuse to participate in the follow-up visit. In the present study ‘optimal outcome’ means that individuals who had ‘recovered’ from ASD. We defined optimal outcome as: no longer met diagnostic criteria for ASD after our diagnosis assessment. Data were analyzed in 2021 October.

### MRI acquisition and data preprocessing

All brain imaging was performed on a 3.0-T imaging system (Philips, Achieva, TX, Best, The Netherlands) at the Tibet Chengban Branch of Sichuan University West China Hospital. Three-dimensional T1 images were acquired using a spoiled gradient recalled echo planar imaging sequence (repetition time, 8.2 ms; echo time, 3.8 ms; flip angle, 7°; slice thickness, 1 mm; field of view, 256 mm × 256 mm; matrix size, 256 × 256; voxel size, 1 × 1 × 1 mm^3^). dMRI scans were acquired using a two-dimensional diffusion-weighted echo planar imaging sequence [repetition time, 10,295 ms; echo time, 91 ms; field of view, 128 × 128 mm; voxel size, 2 × 2 × 2 mm^3^; matrix size, 128 × 128; slice thickness = 2.0 mm, gap = 0 mm, slice number = 75, and slice order = interleaved; 32 gradient direction diffusion-weighted images with b = 1000 s/mm^2^ and one minimally diffusion-weighted scan (the B0 image)].

The dMRI data were processed using FSL^42^. The data were corrected for motion/eddy currents and non-brain tissues were removed. Scans were excluded if they had rotations or translations larger than 3 mm. Groups were compared on FA, MD, RD, and AD using two approaches provided in FSL: Tract-Based Spatial Statistics (TBSS)^12^ and Global Probabilistic Tractography^13^.

### TBSS analysis

Voxelwise analyses were subsequently conducted using TBSS, with mean FA skeleton created by aligning each participant’s FA image to the Montreal Neurological Institute (MNI) 152 space and FA threshed at 0.2 to suppress areas of low fractional anisotropy. Secondary dMRI metrics were analyzed using the mean FA skeleton. Multiple comparison correction was performed using TFCE with identical settings (5000 permutations) in the Randomize program as for the TBSS and further analyses. The p-value threshold for statistical maps was set to 0.05, with TFCE FWE fully corrected.

### Probabilistic tractography

Separately, after quality control, a probabilistic tractography was fitted for each voxel using BEDPOSTX (the probabilistic distribution of diffusion parameters at each voxel was built up by Bayesian estimation of diffusion parameters). First, based on the protocols described by^43,44^, we worked to define standard space “seed”, “target”, “stop” and “exclusion” ROIs, and mapped in individual native space based on anatomical landmarks. Then, tractography was performed for each subject and for each structure in native space with final set consisting of 27 tracts^45^. Low probability voxels were rejected at a threshold of 0.5%, similar to previous study. Only those reconstructed single trajectories present in 70% of the participants in each group were left for subsequent analysis. For each individual separately, we created an average fiber bundle at group level for the 27 major fiber tracts. As a final step, tract density images in subject native space were warped to common space, and then dMRI metrics were exported and averaged across the whole fiber tract for further analysis.

### Statistical analysis

We applied propensity score matching (PSM) to balance the IQ, age, and gender characteristics of the groups in order to avoid bias. We utilized the most common PSM approach of Nearest Neighbor Matching with a 1:1 matching ratio. PSM was performed using the SPSS plug-in psmatching 3.04. After PSM, the categorical and dimensional analysis were performed. A one-way ANOVA was used for each statistic, with post-hoc pairwise group comparisons. The Boneferroni multiple comparison test was employed for multiple comparisons. Pearson correlation was used to explore the association with dMRI metrics and autism symptoms. Multivariate logistic regression analysis was conducted by fitting a logistic regression model to identify neuroimaging risk factors for ASD with ‘optimal outcome’. A statistically significant difference was defined as p < 0.05 (two-tailed).

### Ethics approval and consent to participate

This study was approved by the ethics committee of Medical Ethical Committee of West China Hospital of Sichuan University, and written informed consent was obtained from all participants in accordance with the Declaration of Helsinki.

## Results

### Participants' demographics before and after PSM

Using PSM, ASD+ and TDC groups were matched on age, gender, and IQ to ASD− group. Each group (ASD−, ASD+, and TDC) had a total of 30 participants. (See eFigure 1). As demonstrated in the Table 1, there were no significant differences among groups on age, gender, and IQ (p > 0.05). At baseline, there was a difference in ADIR scores between ASD− and ASD+ (p = 0.003 before matching; p = 0.001 after matching), but not in ADOS scores (p > 0.05). At time 2, the ADOS scores of ASD+ and ASD− individuals were significantly different (p < 0.001) before and after matching. Prior to PSM, the rate of comorbidity was higher in the ASD+ group (16.67%) than in the ASD− group (41.98%). While the results showed that there was no significant difference on the comorbidity rate between ASD+ and ASD− groups after PSM.

**Table 1:** Demographics characteristics of participants before and after propensity score matching.

	Before matching					After PSM				
	ASD− (N = 30)	ASD+ (N = 81)	TDC (N = 72)	F/chi-square	p	ASD− (N = 30)	ASD+ (N = 30)	TDC (N = 30)	F/chi-square	p
T1 age	8.03 (3.95)	8.24 (3.24)	9.00 (2.71)	1.50	0.226	8.03 (3.95)	7.38 (2.95)	8.46 (2.66)	0.85	0.430
Sex (F/M)	1:29	9:72	24:48	667.67	< 0.001***	1:29	1:29	2:28	0.52	0.768
IQ	89.71(18.02)	86.09(19.67)	103.82(15.84)	19.39	< 0.001***	89.71(18.02)	87.47(20.01)	94.42(15.09)	1.19	0.310
T1ADIR	34.76(14.35)	45.15 (14.89)	–	9.29	0.003**	34.76 (14.35)	41.93(10.36)	–	17.32	< 0.001***
T1ADOS	17.52 (5.30)	18.26 (5.13)	–	0.31	0.578	17.52 (5.30)	18.83 (4.39)	–	1.49	0.227
T2ADOS	1.90 (2.38)	14.24 (6.93)	–	90.07	< 0.001***	1.90 (2.38)	13.63 (6.20)	–	93.93	< 0.001***
T2 comorbid	5	34	–	6.153	0.013*	5	11	–	3.068	0.080*

*p < 0.05; **p < 0.01; ***p < 0.001. *ASD−* ASD with optimal outcome, *ASD+* ASD with persistent symptoms, *TDC* typical development controls, *PSM* Propensity Score Matching, *IQ* intelligent quotient, *T1* baseline timepoint, *T2* the follow up timepoint with 4 years after the first assessment, *T2* comorbid: the number of patients who were comorbited with other psychiatric disorders (ADHD, anxiety, depression, and etc.) at follow-up.

### TBSS analysis

**Figure 2:**
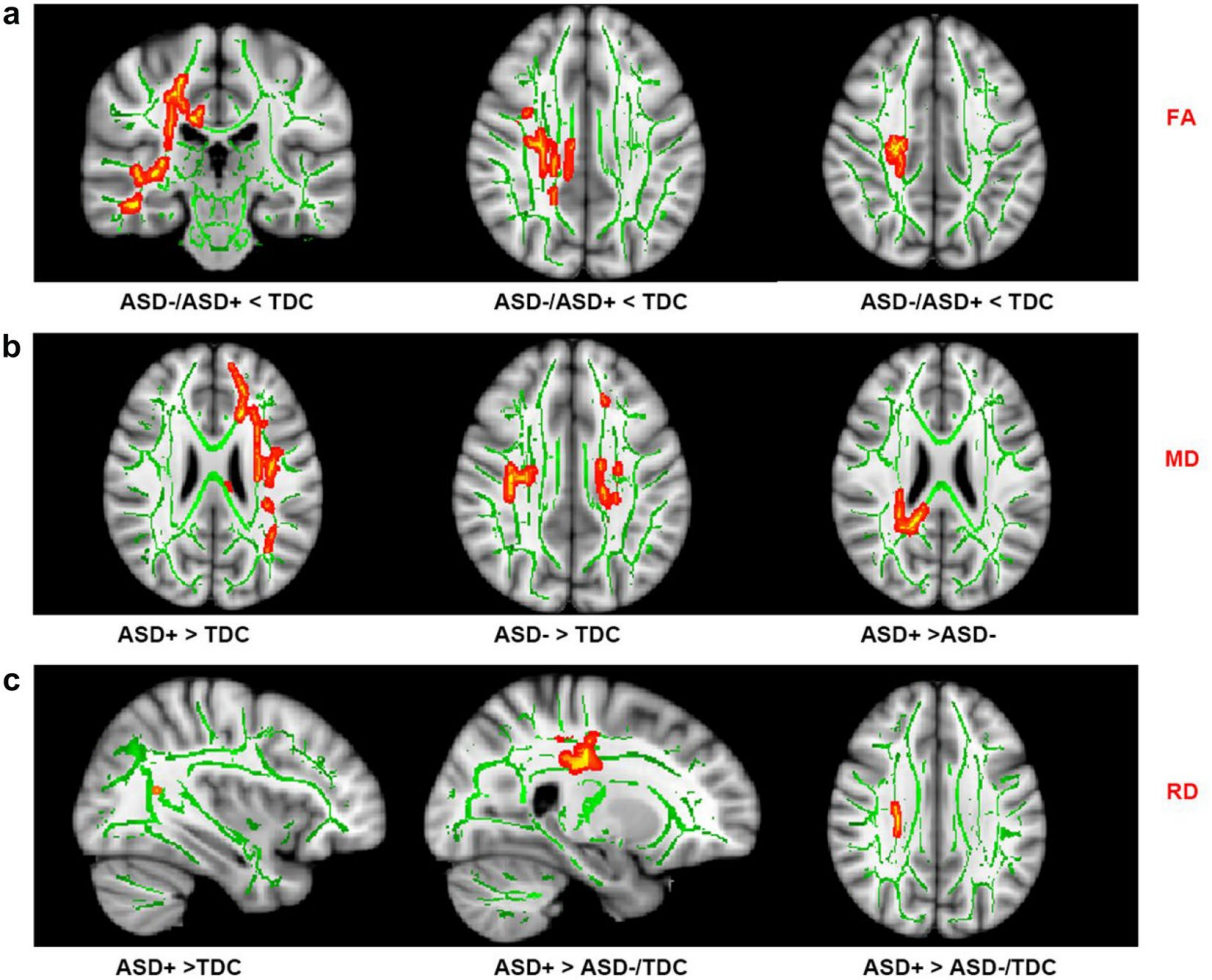
TBSS results among ASD children with optimal outcomes, ASD with persistent symptoms, and controls. TBSS results within the JHU White-Matter Tractography Atlas are reported and labeled accordingly (TFCE FWE corrected, p < 0.05). ANOVA results were symbolled in red. (**a**) FA metrics: ASD−/ASD+  < TDC: Superior longitudinal fasciculus, Inferior longitudinal fasciculus, Forceps major; Cingulum; Corticospinal tract; Inferior fronto-occipital fasciculus; Anterior thalamic radiation; (**b**) MD metrics: ASD+  > TDC: Anterior thalamic radiation; Corticospinal tract L; Cingulum; Inferior fronto-occipital fasciculus L; Superior longitudinal fasciculus; ASD− > TDC: Anterior thalamic radiation R; Corticospinal tract R; Cingulum; Forceps major; Inferior fronto-occipital fasciculus R; Inferior longitudinal fasciculus R; Superior longitudinal fasciculus R; ASD+  > ASD−: Anterior thalamic radiation; Corticospinal tract L; Cingulum L; Forceps major; Inferior fronto-occipital fasciculus L; Inferior longitudinal fasciculus L; Superior longitudinal fasciculus; Uncinate fasciculus L; (**c**) ASD+  > TDC: RD metrics: Inferior longitudinal fasciculus R; Superior longitudinal fasciculus R; ASD+  > ASD−/TDC: Anterior thalamic radiation; Corticospinal tract L; Cingulum L; Inferior fronto-occipital fasciculus L; Inferior longitudinal fasciculus L; Superior longitudinal fasciculus; Uncinate fasciculus L.

We performed TBSS analysis to compare the differences among groups before and after PSMatching. Firstly, prior to PSMatching, the TBSS results showed decreased FA in extensive brain regions (bilateral anterior thalamic radiation (ATR), corticospinal tract (CST), cingulum, inferior fronto-occipital fasciculus, superior longitudinal fasciculus (SLF), and forceps major) in ASD+ versus TDC group. The results also indicated that the ASD− group exhibited reduced FA in the bilateral ATR, CST, cingulum, forceps major; and the right hemisphere of inferior fronto-occipital fasciculus, inferior longitudinal fasciculus (ILF), and SLF, when compared to TDC. However, we did not identify any significant differences in brain area between the ASD− and ASD+ groups, which may be due to confounding variables (unmatched IQ, symptom severity, and comorbidity). (Depicted in eFigure 2) After PSMatching, ANOVA results showed the differential brain areas were SLF, ILF, and extending to ATR, CST, and cingulum (Fig. 2). Children with ASD exhibited reduced FA, and no significant difference between ASD− and ASD+ was found. (Fig. 2a) For MD, results additionally showed increased MD in the SLF, and the left ILF extending to left ATR, CST, and cingulum in ASD+ compared to ASD−. (Fig. 2b) The AD metrics showed no group differences. ASD+ showed increase RD than TDC in the bilateral SLF, ILF, and extending to ATR, CST, and cingulum. ASD+ also showed increase RD than ASD− in the SLF, and the left ILF extending to the left ATR, CST, and cingulum. ASD− did not differ from TDC. (Fig. 2c) The subjects who achieved optimal outcome four years later had less severe brain WM microstructure aberration than those who had persistent diagnosis.

**Figure 3:**
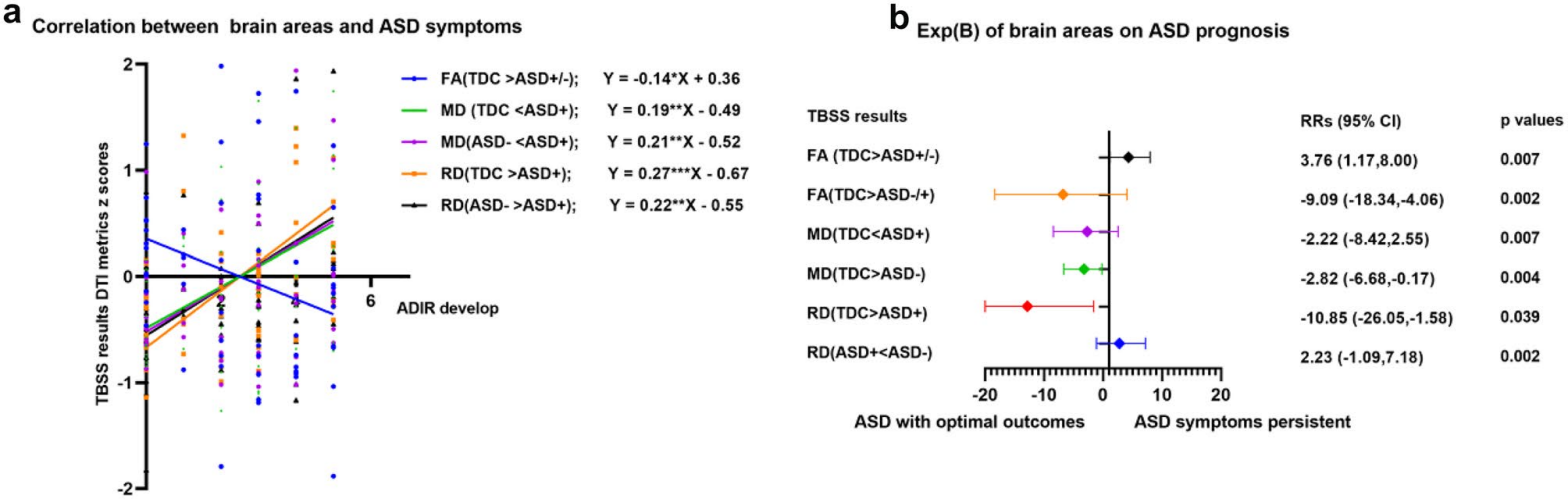
Brain-symptoms data analyses. *p < 0.05; **p < 0.01; ***p < 0.001. (**a**) Statistically significant correlations between dMRI metrics and ADI-R develop subscale. Dotted lines represent the r value for those correlations. (**b**) Forest plot of the multivariable regression analysis results.

Correlation and multiple logistic analysis were used to explore the connections between neuroimaging and symptoms in ASD. The ADI-R develop subscale and the FA had a significant negative association (r = − 0.14, p = 0.03) whereas the MD/RD correlated positively (p < 0.05, see Fig. 3a). Figure 3b shows the findings of multiple logistic analysis. The metrics left in the regression model were depicted. Bilateral SLF, ILF, and extending to ATR, CST, and cingulum FA decreases (ASD+/ASD− < TDC) were associated with ASD with persistent diagnosis (EX(B), 3.76; 95% CI 1.17–8.00; p = 0.007). The right lateral of SLF, ILF, inferior fronto-occipital fasciculus (IFOF), and extending to right ATR, CST, and Cingulum MD decreases (TDC > ASD−) were protective prediction of ASD with persistent diagnosis (EX(B), − 2.82; 95% CI − 6.68 to − 0.17; p = 0.004). The right lateral of SLF and ILF (TDC > ASD+) RD decreases were protective prediction of ASD with persistent symptoms (EX(B), − 10.85; 95% CI − 26.05 to − 1.58; p = 0.002. The association fibers (SLF, ILF, IFOF) and cingulum, particularly in the left lateral, were found to be related with autistic symptoms.

### Probabilistic tractography

**Figure 4:**
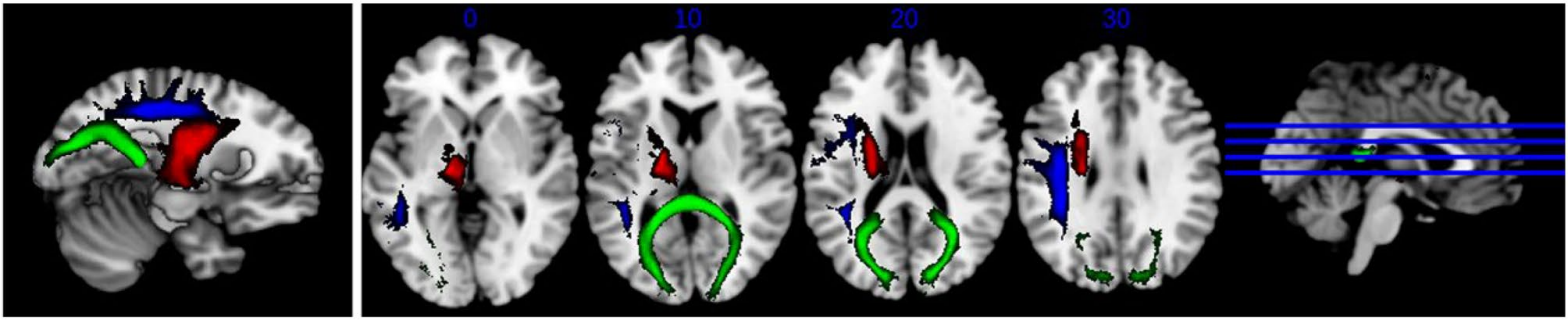
A 3D reconstruction of the three fiber bundles. The red represented the Superior thalamic radiation _L; The blue fiber represented the Superior longitudinal fasciculus_ L; The green represented the Forceps major.

After matching participants, individual probabilistic tractography of the 27 major WM tracts was successfully obtained. ANOVA with post-hoc Boneferroni correction was used to compare the ASD−, ASD+, and TDC groups. Three damaged fiber bundles were displayed in Fig. 4 in ASD: Superior thalamic radiation _L (STR_L), SLF_L, and Forceps major (Fma).

**Figure 5:**
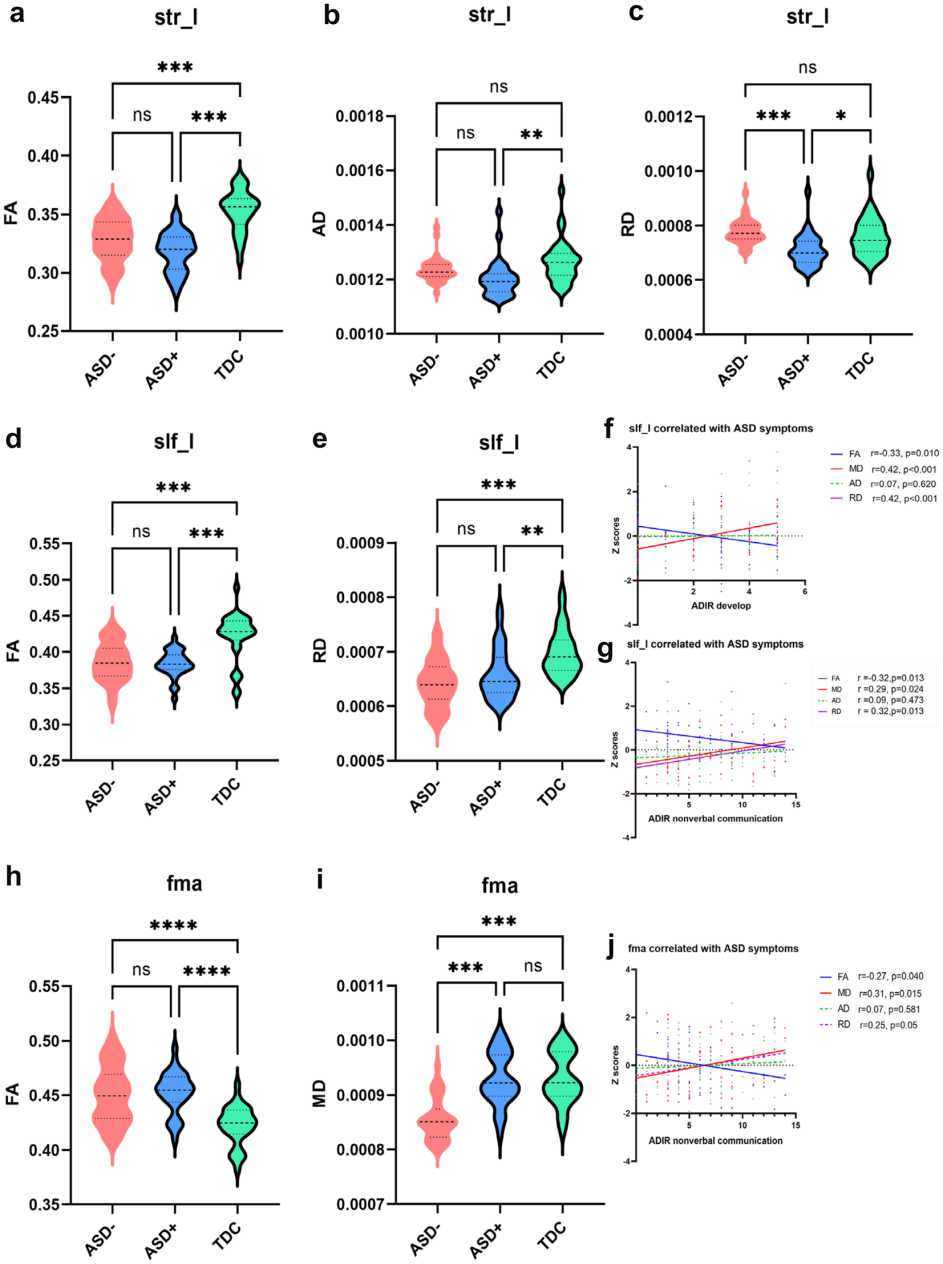
Varied fiber tract of ASD via probabilistic tractography. *p < 0.05; **p < 0.01; ***p < 0.001. *ASD−* ASD with optimal outcome, *ASD+* ASD with persistent symptoms, *TDC* typically developing controls, *str_l* left superior thalamic radiation, *slf_l* left superior longitudinal fasciculus, *fma* forceps major.

For further analysis, dMRI metrics were computed for each of the reconstructed tracts by averaging the voxel values along each fiber bundle. ANOVA revealed that the three groups differed in terms of STR L, SLF L, and Fma (see Fig. 5). As to STR_L, ASD− and ASD+ exhibited lower FA than TDC (p < 0.001), but ASD with different prognosis had no significant difference (p > 0.05, Fig. 5a). As AD, the STR L abnormality was shown in ASD+ (p = 0.002), but not in ASD− (p > 0.05, Fig. 5b). ASD+ exhibited a lower RD than ASD− (p < 0.001) and TDC (p = 0.049) (Fig. 5c). It suggested ASD+ was more severe than ASD-. STR_L had no significant correlation with autism symptoms. Figure 5d and e showed that ASD− and ASD+ exhibited reduced FA and RD in SLF_L (p < 0.05). Correlation analysis showed FA was negatively correlated with ADIR develop/ nonverbal communication subscale, and MD, RD were positively correlated with ADIR develop/ nonverbal communication (Fig. 5f–g). The results revealed an association between SLF L fiber bundle impairment and autism symptoms. Regarding Fma tract; ASD+ and ASD− had greater FA than TDC (p < 0.001, Fig. 5h). ASD− exhibited decreased MD than ASD+ and TDC (p < 0.001, Fig. 5i). Further correlation analysis revealed that FA was negatively correlated with ADIR-nonverbal communication subscale, whereas MD was positively correlated (Fig. 5j). ASD− showed the most intact integrity in Fma among the three groups.

Table 2 shows fiber tracts associated with ASD prognoses in four years. The total collinearity amongst dMRI metrics was significant. We separately examine the binary logistic analysis of ASD prognoses with fiber bundles using FA, MD, AD, RD. The STR_L fiber tract was observed to be associated with optimal outcome (FA: EX(B), 6.25; 95% CI 2.50–15.63; p < 0.001; inversely, for MD, AD, RD, EX(B) was 0.10, 0.02,0.17 separately). The STR_L tract integrity was a predictive factor of optimal outcome. Fma MD increasement was a predictive factor of optimal outcome (MD: EX(B), 28.46; 95% CI 4.33–187.10; p < 0.001). It indicated that Fma fiber integrity impairment was a predictive factor of ASD with optimal outcome.

**Table 2:** Binary logistic regression model to predict ASD with optimal outcome in 4-years follow-up with 27 main fiber bundles.

DTI metrics	Variables	Estimate	Standard error	Wald	p value	Exp (B)	95% CI Exp (B)	
							Lower	Upper
FA total model variance explained, r^2^ = 0.494	str_l	1.83	0.47	15.32	< 0.001***	6.25	2.50	15.63
	Constant	0.26	0.34	0.61	0.436	1.30	–	–
MD total model variance explained, r^2^ = 0.796	fma	3.35	0.96	12.14	< 0.001***	28.46	4.33	187.10
	str_l	− 2.30	0.71	10.62	0.001**	0.10	0.03	0.40
	Constant	0.50	0.55	0.82	0.364	1.64	–	–
AD total model variance explained, r^2^ = 0.643	ar_l	1.86	0.82	5.17	0.023*	6.40	1.29	31.67
	atr_l	3.60	1.11	10.59	0.001**	36.43	4.18	317.61
	str_l	− 4.13	1.17	12.41	< 0.001***	0.02	0.002	0.16
	Constant	− 0.14	0.43	0.10	0.752	0.87	–	–
RD total model variance explained, r^2^ = 0.74	str_l	− 1.75	0.49	12.86	< 0.001***	0.17	0.07	0.45
	Constant	− 0.18	0.32	0.33	0.564	0.83	–	–

*p < 0.05; **p < 0.01; ***p < 0.001. *FA* fractional anisotropy, *MD* mean diffusivity, *AD* axial diffusivity, *RD* radial diffusivity, *str_l* the left superior longitudinal fasciculus, *fma* forceps major, *ar_l* the left acoustic radiation, *atr_l* the left anterior thalamic radiation.

## Discussions

This was the first study to compare ASD with persistent diagnosis versus ASD with optimal outcome in terms of brain white matter abnormalities. The ASD cohort had MRI scans and symptom evaluation during the initial visit, and their symptoms were reassessed four years later. In addition to TBSS, we applied probabilistic tractography to reduce the impact of crossing fibers and construct more precise white matter tracts. The most obvious result of the TBSS and probabilistic tractography analyses was the confirmation of the SLF L white matter abnormalities in ASD, which is consistent with prior studies^16,17^. Moreover, we identified a correlation between the SLF L anomaly and the severity of ASD in children with ASD. However, no significant difference between ASD− and ASD+ was identified. Statistical analysis revealed no evidence of a connection between SLF and ASD prognosis following a 4-year follow-up period. Another interesting finding is that, the projection fibers such as ATR, CST, also showed abnormalities in ASD by TBSS. Further analysis with probabilistic tractography identified STR L abnormalities between ASD with different prognosis, indicating that ASD with persistent diagnosis showed more severe WM abnormalities than ASD with optimal outcome. Logistic analysis revealed that the integrity of the STR L tract was a predictor factor of optimal outcome.

Children with ASD exhibit lower FA and RD on SLF L, and the FA is adversely connected with nonverbal communication problems associated with ASD. The SLF connects mirror neurons and is thought to be necessary for gestural communication and language development. It was recently included in a five-level anatomical model of social communication^46^. SLF can be divided into three parts, SLF I-II-III, SLF I/II is involved in the perception of visual space, whereas SLF III is involved in language processing^47^; both of which are cognitive processes associated with ASD. Previously published dMRI studies indicated that ASD patients have poor SLF fiber integrity^17,48^. Our cohort study is the first to utilize TBSS and probabilistic tractography to explore the brain WM architecture in ASD with optimal outcome. Regardless of the outcome of the children, ASD displayed a similar SLF abnormality. Our data imply that while abnormalities in SLF L may reflect the degree of communicative difficulties in ASD, they are not connected with four-year prognosis. This may support to the 'neural normalization' hypothesis: the neuroimaging of ASD with optimal outcome is similar to normal children^34,35^. ASD behavioral normalization is a process of neuron remodeling, as opposed to exhibiting different neuroimaging pathways from the outset. The current study emphasized the functional significance of this fiber, albeit further MRI rescans were required to assess the developing trajectory of SLF in ASD.

Concerning STR L, the FA value of ASD+ group was lower than ASD-, indicating a severe STR L fiber bundle abnormality in children with persistent ASD symptoms. The thalamus is engaged in a variety of sensory and cognitive circuits in healthy individuals. The thalamo-cortical circuit is a critical information processing system^49^ Indirectly, prior research implicating the thalamus in the pathogenesis of autism^15,50^ supports our findings. Several thalamocortical fibers abnormalities had been found in ASD: the anterior thalamic radiation, major projection from the thalamus to the frontal cortex^49,51–53^; the superior thalamic radiation, projection fiber from the ventral posterior nuclei of the thalamus to the somatosensory area in the post central gyrus of the parietal cortex^15,54^; and the posterior thalamic radiation, projection fiber from the posterior part of the thalamus to the occipital cortex^15^. Our cohort analysis revealed that ASD with optimal outcome is associated with distinct neuroimaging abnormalities in STR L, and STR-L integrity is proven to be a predictor of ASD with optimal outcome after four years. Verification of this result will require a larger sample size and a longer period of follow-up.

The splenium of the corpus callosum connects with the occipital lobes to form the Forceps major (Fma). The Fma plays roles in several ASD related cognitions, such as semantic processing^55^, visuospatial cognition^55^, and language comprehension^56^. The results of the present study demonstrate that the ASD− showed higher FA (and lower MD) than ASD+ and TDC. As the FA values of the traced antegrade fibers were extracted to indicate the integrity of the epicenter white matter. It suggested that the ASD with optimal outcome had the most intact WM integrity among the three groups. The compensation theory may explain this phenomenon^36^. This finding contradicts prior research indicating that ASD had a reduced FA in Fma^57^. Similarly, previous tractography studies have also reported inconsistent results indicating that individual with ASD did not exhibit WM abnormality in Fma^18,58^. The inconsistent results may be due to the heterogeneity of ASD. Moreover, Fma integrity impairment (MD increased) predicted ASD with optimal outcome. The finding was not replicated by other dMRI metrics in this study, thus the conclusion must be interpreted cautiously.

The term ’optimal outcome’ referred to individual with ASD history who no longer fit the diagnostic criteria for ASD^26,27^. Our cohort's recovery rate is 27%, which is consistent with prior research (1–32%)^33,59^. We observed a high recovery rate of ASD, maybe because we included all high-functioning autism. Additionally, our criteria for ‘optimal outcome’ are clinical judgement, further objective measures such as social adaptive scales may be adopted in the future. The concept of ‘optimal outcome’ has led to a controversial topic: can ASD be cured? Longitudinal cohort studies of ASD have consistently found that some patients with ASD no longer meet the diagnostic criteria for ASD in adulthood^26,27,60,61^. Further analyses^26,27,62,63^ have identified common characteristics in ASD with ‘optimal outcome’: normal intelligence, impaired social cognition, unsatisfied intimate relationships, poor neurocognitive functioning. Studies have also identified some risk factors associated with ASD prognosis, including: intelligence^64–66^, language ability^67^, symptom severity^68^, gender^69^, etc. The effect of symptom severity on prognosis is well established^68^. In this study, there was no difference in baseline symptom severity between ASD+ and ASD− groups, allowing us to evaluate the effects of neuroimaging features on ASD prognosis. Additional research is required to determine the predictors of ASD prognosis.

## Limitations

This study contains some limitations. To begin, our sample was limited to individuals with high-functioning ASD to ensure compliance with scanner guidelines. Additionally, we excluded ASD with comorbidities from this investigation, which may limit its generalizability to the broader clinical ASD community. The rigorous inclusion/exclusion criteria were implemented to avoid bias; additional research was required to include a larger sample size with stratified analyses. Second, our original study sample size was moderate, which likely increased our type II error rate. Third, a four-year follow-up is insufficient, as parts of ASD with persistent diagnosis will develop to adapt to social life over time. Larger samples and longer follow-up periods will enable future research on WM microstructure in ASD with optimal outcome. Finally, because we did not rescan the participants at timepoint 2, we cannot assess developing trajectory of neuroimaging anomalies in ASD.

## Conclusions

We found that children with ASD showed significant differences in brain WM microstructure in SLF_L and STR_L fibers. STR_L fiber integrity differed between optimal outcome group and persistent diagnosis group, and STR_L was a predictor of behavioral normalization. Our findings may help understand the mechanisms of optimal outcome in ASD and develop therapeutic targets to overcome the new conundrum of heterogeneity impeding ASD pathogenesis research.

## Supplementary Information

Supplementary Information.

## Data Availability

The datasets used and/or analyzed during the current study are available from the corresponding author on reasonable request.
