# Supplementary material for: Brain white matter microstructure abnormalities in children with optimal outcome from autism: a four-year follow-up study
Source: Sci Rep. 2022 Nov 23;12:20151. doi: 10.1038/s41598-022-21085-8 (PMC9684497; doi:10.1038/s41598-022-21085-8)
Supplement: Supplementary file 1 — Supplementary Information. [file 41598_2022_21085_MOESM1_ESM.docx]

**Supplementary materials**


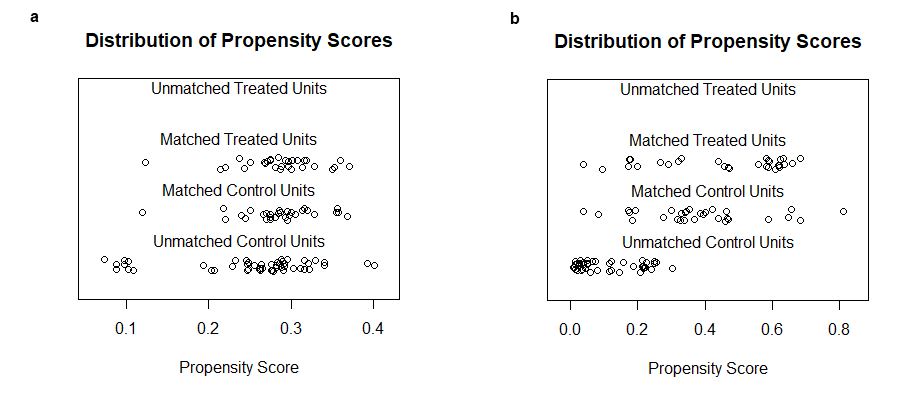


**eFigure 1: PSM results**

Note: **eFigure 1-a** demonstrated the PSM results of the ASD with persistent symptoms group (ASD+) when matched with ASD with optimal outcome group (ASD-). Unmatched treated units represented the ASD- participants that were unmatched (N=0); matched treated units represented the ASD- participants that were matched(N=30); Matched control units represented the ASD+ children that were matched(N=30); Unmatched control units represented the ASD+ children that were unmatched (N=51); **eFigure 1-b** demonstrated the PSM results of the typically developing controls group (TDC) when matched with ASD-. Unmatched treated units represented the ASD- participants that were unmatched (N=0); matched treated units represented the ASD- participants that were matched(N=30); Matched control units represented the TDC that were matched(N=30); Unmatched control units represented the TDC that were unmatched (N=42).


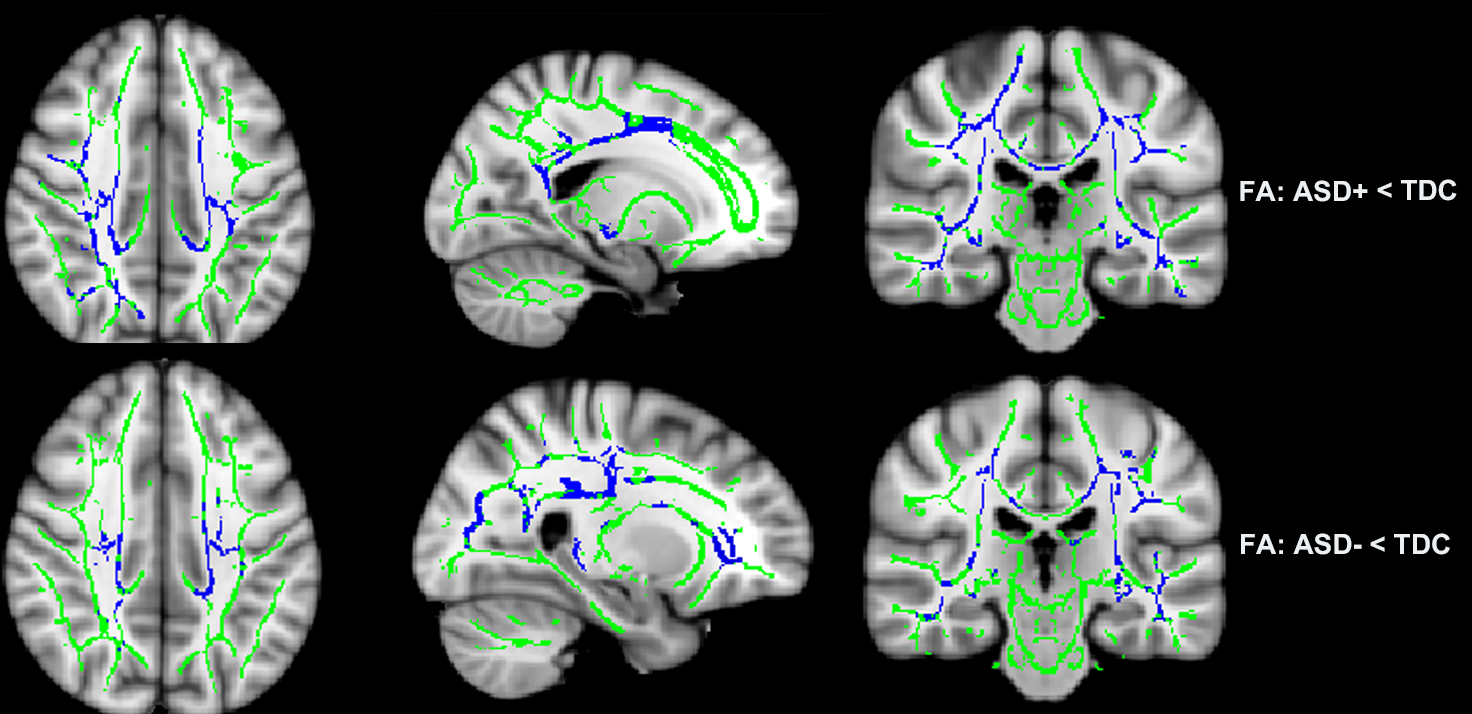


**eFigure 2: TBSS results between ASD-, ASD+, and TDC groups before PSMatching**

**Note:** ASD-: ASD with optimal outcome; ASD+: ASD with persistent symptoms; TDC: typical development controls; FA: fractional anisotropy.

First row: FA ASD+ < TDC: bilateral anterior thalamic radiation (ATR), corticospinal tract (CST), cingulum, inferior fronto-occipital fasciculus, superior longitudinal fasciculus (SLF), forceps major; Second row: FA ASD- < TDC: the bilateral ATR, CST, cingulum, forceps major; and the right hemisphere of inferior fronto-occipital fasciculus, inferior longitudinal fasciculus, and SLF

**eTable 1. TBSS results of dMRI metrics in differential brain regions among ASD-, ASD+, and TDC groups**

| Post-hoc | metrics | Brain region | Cluster voxels | ASD+ | ASD- | TDC | p value(post-hoc) |
| --- | --- | --- | --- | --- | --- | --- | --- |
| ASD-<TDC | FA | Anterior thalamic radiation R  Corticospinal tract  Cingulum R  Inferior fronto-occipital fasciculus  Superior longitudinal fasciculus R  Superior longitudinal fasciculus R | 940 | 0.48(0.04) | 0.49(0.04) | 0.52(0.04) | 0.001 |
| ASD->ASD+ | FA | Anterior thalamic radiation R  Cingulum R  Forceps minor  Uncinate fasciculus R | 137 | 0.25(0.03) | 0.27(0.04) | 0.28(0.04) | <0.0001 |
| ASD+<TDC | FA | Anterior thalamic radiation  Corticospinal tract  Cingulum  Forceps major  Inferior fronto-occipital fasciculus  Inferior longitudinal fasciculus  Superior longitudinal fasciculus  Uncinate fasciculus  Superior longitudinal fasciculus | 55950 | 0.45(0.02) | 0.46(0.03) | 0.47(0.02) | 0.001 |
| ASD->TDC | MD | Anterior thalamic radiation R  Forceps minor  Inferior fronto-occipital fasciculus R  Uncinate fasciculus R | 812 | 0.000834 (0.000038) | 0.000858 (0.000071) | 0.000816 (0.000039) | 0.10 |
| ASD->TDC | MD | Cingulum L  Forceps minor  Inferior fronto-occipital fasciculus L  Inferior longitudinal fasciculus L  Superior longitudinal fasciculus L  Uncinate fasciculus L | 507 | 0.000880 (0.000052) | 0.000905 (0.000076) | 0.000859 (0.000073) | 0.034 |
| ASD->TDC | MD | Corticospinal tract  Cingulum R  Inferior fronto-occipital fasciculus R  Superior longitudinal fasciculus R | 170 | 0.000777 (0.000034) | 0.000792 (0.000042) | 0.000764 (0.000029) | 0.014 |
| ASD->TDC | MD | Anterior thalamic radiation  Corticospinal tract L  CingulumL  Inferior fronto-occipital fasciculus L  Superior longitudinal fasciculus | 149 | 0.000810 (0.000045) | 0.000830 (0.000057) | 0.000795 (0.000048) | 0.032 |
| ASD-<ASD+ | MD | Anterior thalamic radiation  Corticospinal tract L  Cingulum  Forceps major  Inferior fronto-occipital fasciculus L  Superior longitudinal fasciculus  Uncinate fasciculus L | 8041 | 0.000864 (0.000049) | 0.000824 (0.000043) | 0.000846 (0.000046) | 0.005 |
| ASD+>TDC | MD | Anterior thalamic radiation R  Corticospinal tract R  Cingulum  Forceps major  Inferior fronto-occipital fasciculus R  Inferior longitudinal fasciculus R  Superior longitudinal fasciculus R | 773 | 0.000815 (0.000058) | 0.000791 (0.000047) | 0.000786 (0.000037) | 0.034 |
| ASD+<TDC | RD | Anterior thalamic radiation L  Corticospinal tract L  Forceps minor  Inferior fronto-occipital fasciculus L  Superior longitudinal fasciculus L  Uncinate fasciculus L | 362 | 0.001163 (0.000051) | 0.001175 (0.000043) | 0.001210 (0.000066) | 0.046 |
| ASD+<TDC | RD | Anterior thalamic radiation L  Cingulum L  Forceps minor | 343 | 0.001533 (0.000077) | 0.001594 (0.000109) | 0.001558 (0.000079) | 0.006 |
| ASD+<ASD- | RD | Anterior thalamic radiation  Corticospinal tract L  Cingulum L  Forceps minor  Inferior fronto-occipital fasciculus L  Inferior longitudinal fasciculus L  Superior longitudinal fasciculus  Uncinate fasciculus L | 4759 | 0.001389 (0.000091) | 0.001456 (0.000098) | 0.001404 (0.000056) | 0.004 |

Note: ASD-: ASD with optimal outcome; ASD+: ASD with persistent symptoms; TDC: typical development controls; FA: fractional anisotropy, MD: mean diffusivity, RD: radial diffusivity.

**eTable 2. Probabilistic tractography results in differential WM tracts among ASD-, ASD+, and TDC groups**

| WM tract | metrics | ASD+ | ASD- | TDC | F | p | Eta |
| --- | --- | --- | --- | --- | --- | --- | --- |
| STR L | FA | 0.33 (0.02) | 0.35 (0.02) | 0.36 (0.02) | 22.068 | <0.001 | 0.337 |
|  | AD | 0.001203 (0.000068) | 0.001237 (0.000048) | 0.001266 (0.000078) | 7.005 | 0.002 | 0.139 |
|  | RD | 0.000711 (0.000060) | 0.000778 (0.000046) | 0.000760 (0.000072) | 10.108 | <0.001 | 0.189 |
| SLF L | FA | 0.33 (0.03) | 0.38 (0.02) | 0.39 (0.03) | 67.368 | <0.001 | 0.608 |
|  | RD | 0.000659 (0.000047) | 0.000642 (0.000043) | 0.000700 (0.000043) | 13.218 | <0.001 | 0.233 |
| Fma | FA | 0.45 (0.02) | 0.45 (0.03) | 0.44 (0.03) | 18.809 | <0.001 | 0.302 |
|  | MD | 0.000929 (0.000048) | 0.000855 (0.000039) | 0.000930 (0.000047) | 27.973 | <0.001 | 0.391 |

Note: ASD-: ASD with optimal outcome; ASD+: ASD with persistent symptoms; TDC: typical development controls; STR L: left superior thalamic radiation; SLF L: left superior longitudinal fasciculus; Fma: forceps major.
